# Genetic Variation Is the Major Determinant of Individual Differences in Leukocyte Endothelial Adhesion

**DOI:** 10.1371/journal.pone.0087883

**Published:** 2014-02-10

**Authors:** Michael A. Grassi, Vidhya Rao, Kathryn P. Winkler, Wei Zhang, Joseph D. Bogaard, Siquan Chen, Bonnie LaCroix, Divya Lenkala, Jalees Rehman, Asrar B. Malik, Nancy J. Cox, R. Stephanie Huang

**Affiliations:** 1 Department of Ophthalmology & Visual Sciences, University of Illinois at Chicago, Chicago, Illinois, United States of America; 2 Department of Pediatrics, University of Illinois at Chicago, Chicago, Illinois, United States of America; 3 Cellular Screening Center, Institute for Genomics and Systems Biology, University of Chicago, Chicago, Illinois, United States of America; 4 Department of Pharmacology, University of Illinois at Chicago, Chicago, Illinois, United States of America; 5 Section of Genetic Medicine, University of Chicago, Chicago, Illinois, United States of America; 6 Section of Hematology/Oncology, University of Chicago, Chicago, Illinois, United States of America; University Medical Center Utrecht, Netherlands

## Abstract

**Objective:**

To determine the genetic contribution to leukocyte endothelial adhesion.

**Methods:**

Leukocyte endothelial adhesion was assessed through a novel cell-based assay using human lymphoblastoid cell lines. A high-throughput screening method was developed to evaluate the inter-individual variability in leukocyte endothelial adhesion using lymphoblastoid cell lines derived from different donors. To assess heritability, ninety-two lymphoblastoid cell lines derived from twenty-three monozygotic twin pairs and twenty-three sibling pairs were compared. These lymphoblastoid cell lines were plated with the endothelial cell line EA.hy926 and labeled with Calcein AM dye. Fluorescence was assessed to determine endothelial cell adhesion to each lymphoblastoid cell line. Intra-pair similarity was determined for monozygotic twins and siblings using Pearson pairwise correlation coefficients.

**Results:**

A leukocyte endothelial adhesion assay for lymphoblastoid cell lines was developed and optimized (CV = 8.68, Z′-factor = 0.67, SNR = 18.41). A higher adhesion correlation was found between the twins than that between the siblings. Intra-pair similarity for leukocyte endothelial adhesion in monozygotic twins was 0.60 compared to 0.25 in the siblings. The extent to which these differences are attributable to underlying genetic factors was quantified and the heritability of leukocyte endothelial adhesion was calculated to be 69.66% (p-value<0.0001).

**Conclusions:**

There is a heritable component to leukocyte endothelial adhesion. Underlying genetic predisposition plays a significant role in inter-individual variability of leukocyte endothelial adhesion.

## Introduction

Leukocyte endothelial adhesion has been implicated as a key disease process in many conditions of major public health import such as multiple sclerosis, atherosclerosis, inflammatory bowel disease, diabetic retinopathy, malignancy, pulmonary edema, rheumatoid arthritis, and stroke [1], [2]. Attempts to target leukocyte trafficking as a treatment for these conditions have been successful, but often have serious complications given the multiple off-target effects of many of these drugs e.g. prednisone [2], [3]. Leukocyte–endothelial interactions are primarily attributed to cell-surface adhesion molecules including the integrins, members of the IgG superfamily, and selectins. There remain many other, yet undefined, molecules and signaling pathways that mediate leukocyte endothelial adhesion [4]. Improved characterization of the molecular underpinnings of leukocyte endothelial adhesion could have far reaching biomedical impact as it may improve therapeutic targeting and specificity for many common diseases. Genomic studies may have the capability of accomplishing this goal, but require there to be an underlying genetic basis for leukocyte endothelial adhesion.

Corroborating lines of evidence suggest that genetic variability contributes to differences in leukocyte endothelial adhesion. For instance, it has been shown that the expression of cell surface adhesion molecules on endothelial cells varies between individuals [5] and is highly heritable. Rainwater et al. demonstrated that in the setting of TNF-α there is a significant genetic contribution to the expression of E-selectin, VCAM, and ICAM-1 [6]. Moreover, hematologic malignancies like leukemia caused by underlying chromosomal re-arrangements reveal differential gene expression of adhesion molecules resulting in altered endothelial adhesion [7]. For instance, in Burkitt lymphoma endothelial adhesion is significantly decreased [8]. Findings in a rare Mendelian condition, leukocyte adhesion deficiency, also suggest that there is a genetic component to leukocyte endothelial adhesion. Leukocyte adhesion deficiency is an immunodeficiency characterized by severe recurrent bacterial infections [9]. The condition is caused by a lack of leukocyte β-2 integrins that produce an impairment of leukocyte interactions necessary to fight infection. A broad clinical range of leukocyte adhesion deficiency exists that correlates with the severity of the pathogenic mutation. Similarly, individuals with Down's syndrome (Trisomy 21) have a genetic basis for their enhanced adhesive characteristics in comparison to normal subjects [10]. Trisomy 21 leukocytes demonstrate increased expression of CD18 integrin, which is found on chromosome 21 [10]. Taken together these findings suggest that there may be a significant underlying genetic component that contributes to differences in leukocyte endothelial adhesion.

To explore the hypothesis that inter-individual genetic variability contributes to differences in leukocyte endothelial adhesion we assessed adhesion in monozygotic twins and compared them to matched sibling pairs. We quantified the extent to which these differences are attributable to underlying genetic factors [11], [12] to determine the heritability of leukocyte endothelial adhesion.

## Materials and Methods

### Ethics

Participants gave written informed consent. The University of Illinois at Chicago Institutional Review Board approved all protocols and informed consent documents in accordance with federal regulations and the principles expressed in the Declaration of Helsinki.

### Cell lines and Cell culture

Ninety–two lymphoblastoid cell lines (twenty-three monozygotic twin pairs and twenty-three sibling pairs) were purchased from Coriell Institute for Medical Research (http://ccr.coriell.org/) (Table S1). The minimum number of lymphoblastoid cell lines pairs required for the study was estimated as previously described [13]. Monozygotic twin cell lines used in the study were GM14381, GM14382, GM14405, GM14406, GM14414, GM14417, GM14432, GM14433, GM14452, GM14453, GM14467, GM14468, GM14478, GM14479, GM14506, GM14507, GM14520, GM14521, GM14568, GM14569, GM14581, GM14582, GM14408, GM14409, GM14447, GM14448, GM14592, GM14593, GM14474, GM14475, GM14503, GM14504, GM14532, GM14533, GM14535, GM14536, GM14583, GM14584, GM14476, GM14477, GM14480, GM14481, GM14495, GM14496, GM14501, GM14502. The sibling cell lines used in the study were GM7004, GM07012, GM07044, GM7052, GM07343, GM07344, GM11922, GM11923, GM11982, GM11983, GM11985, GM11986, GM11997, GM11999, GM12036, GM12038, GM12104, GM12105, GM12147, GM12149, GM12150, GM12157, GM11834, GM11841, GM12047, GM12048, GM07351, GM07352, GM14666, GM14672, GM14661, GM14662, GM14682, GM14704, GM12328, GM12265, GM11871, GM11872, GM13117, GM13118, GM12276, GM12277, GM07023, GM07059, GM07062, GM07053. Sibling pairs were all comprised of full siblings. Ramos, a Burkitt Lymphoma cell line obtained from ATCC (CRL-1596, ATCC, Manassas, VA) was a kind gift from Dr. Chandran (Rosalind Franklin University, North Chicago, IL). A Trisomy 21 lymphoblastoid cell line, GM03716, was purchased from Coriell cell repositories (Camden, New Jersey). U937, a monocytic cell line, and EA.hy926, a transformed endothelial cell line, were obtained from ATCC (American Type Culture Collection, Manassas, VA). Peripheral blood mononuclear cells were obtained from a healthy control donor.

### Leukocyte-Endothelial Cell Adhesion Assay

Lymphoblastoid cell lines were plated on the endothelial cell line EA.hy926 to test their adhesion. EA.hy926 cells were counted using the Beckman coulter counter, plated at a density of 12,000 cells/well in flat clear bottom black 96-well plates (Corning, Acton, MA), and cultured to confluency for 48 hours. On the day of the assay, lymphoblastoid cell lines or peripheral blood mononuclear cells were suspended in RPMI 1640 with 10% FBS at a concentration of 8×10^5^ cells/mL and incubated with 2 µM Calcein AM for 30 min at 37°C. The cells were collected and subjected to three washes with PBS (1X, Gibco, Life technologies, Grand Island, NY) to remove the free Calcein AM. Calcein AM labeled cells were added at a density of 50,000 cells/well on top of confluent monolayer cultures of EA.hy926 cells in 96-well plates and incubated for 30 min at 37°C. Nonadherant cells were removed by an optimized automated wash protocol adapted using the EP3 liquid handling system. Calcein AM fluorescence in the labeled cells was assessed by the high content imager Acumen at an excitation and emission wavelength of 485/535 nm before and after each wash. Endothelial adhesion for each cell line was determined by measuring the total area of fluorescence in relative fluorescence units (RFU) of each well following the third wash. The (%) remaining RFU/Input was determined by the following formula: [Total area of fluorescence (RFU) following third wash / Total area of fluorescence (RFU) before wash] ×100. A standard curve for lymphoblastoid cell lines, peripheral blood mononuclear cells, and the control cell lines (Trisomy lymphoblastoid cell line, U937, and Ramos) was generated using known concentrations of respective cell lines and fitting to a four parametric logistic curve (Figure S1). The % adherent cells were derived from the standard curve. The leukocyte endothelial adhesion (LEA) of each lymphoblastoid cell line was represented as either (%) of input relative fluorescent units (RFU) or (%) of input cell number.

### Statistical Analysis

A mean, standard deviation, and coefficient of variation (CV) of leukocyte endothelial adhesion for each lymphoblastoid cell line was calculated. Plates included positive and negative controls for quality control. Positive controls were a Trisomy 21 lymphoblastoid cell line that also provided a reference for the control of inter-plate variability. Ramos, a Burkitt lymphoma cell line, served as an additional positive control. The negative controls were EA.hy926 cells alone. A Z′-factor and CV for each plate was determined. A composite Z′-factor was determined to assess the overall robustness of the assay using the equation 
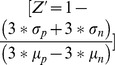
with Trisomy 21 as the positive control and Ramos as the negative control. A Z′-factor of >0.5 is equivalent to a separation of 12 standard deviations between 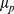 and 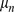.

Intra-pair similarity was determined for monozygotic twins and siblings using the interclass correlation coefficient (ICC). Heritability was estimated by using the twinan90 function in the R package [14]. Sibling pairs were treated as dizygotic twins and heritability 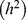
was estimated using the equation, 
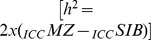
, where r corresponds to the interclass correlation coefficient of twin and sibling pairs respectively [15]. We performed 10,000 simulations by randomizing the samples to generate a null distribution. We estimated an empirical p-value for the heritability. Linear Pearson's correlation coefficient (r) was used to determine the contributions of potential confounders e.g., subject age, gender, cell growth rate, ethnicity and transformation time. Statistical calculations and graphing were performed using Intercooled Stata 8 (Stata Corp., College Station, TX).

## Results

### Adhesion assay development, optimization and validation

**Figure 1:**
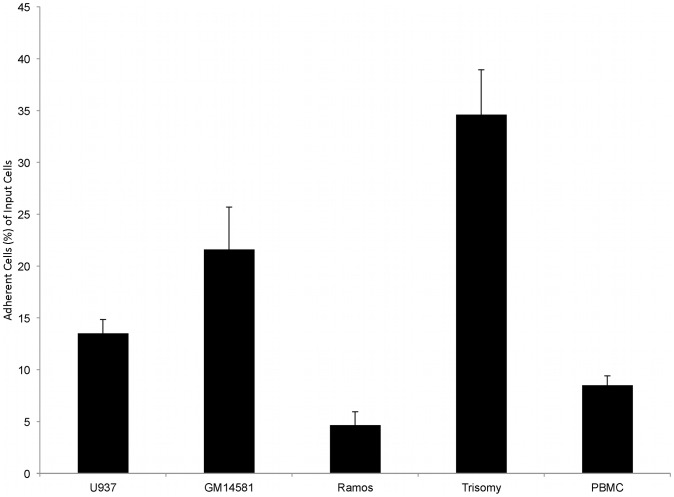
Percentage of adherent cells by sample type. Number of cells is the percentage remaining after three washes. The assay reveals a large dynamic range between the Burkitt lymphoma Ramos cell line (negative control) and the Trisomy 21 lymphoblastoid cell line (positive control). Consistent with other reports, peripheral blood mononuclear cells demonstrated approximately 5–10% adhesion. Percentages were derived based on conversion of relative fluorescent units from a standardized curve.

We developed and optimized a leukocyte endothelial adhesion assay for lymphoblastoid cell lines (CV = 8.68, Z′-factor = 0.67, SNR = 18.41). Specifically, we have successfully converted this assay onto a 96-well plate platform amenable to robotic screening. The advantage of this format is that it affords a high degree of consistency and repeatability between runs (Figure 1). A 96-well plate platform is particularly advantageous for this experiment given the large numbers of samples that were utilized. A total of 8 technical replicates were performed to determine the leukocyte endothelial adhesion of each lymphoblastoid cell line. For a given plate, standard deviations and coefficients of variation significantly less than 10% suggest that there is little variability between replicates of a given cell line. In addition, we calculated a Z′-factor of 0.67 for the assay, suggesting that the assay is excellent from the standpoint of quality control. The Z′-factor is a measure of the statistical effect that assesses the suitability of a particular assay for use in a full-scale high-throughput screen by essentially comparing the range between the positive vs. the negative control samples. In order to control for plate-to-plate variability, samples were standardized based on the adhesion of the Trisomy 21 lymphoblastoid cell line and the adhesion of the U937 monocytic cell line on each plate. For instance, the leukocyte endothelial adhesion of lymphoblastoid cell line GM14581 was tested in three different plates and its ratio of adhesion relative to the Trisomy 21 cell line was 0.64, 0.67 and 0.68 respectively. Standardized coefficients for the same cells (GM14581, Ramos and U937) on different plates did not demonstrate a statistically significant difference (p-value = 0.62).

The assay demonstrates a large dynamic range, with close to a 7-fold difference, evident between the control cell lines, namely the low adhesive Burkitt lymphoma Ramos line and the highly adhesive Trisomy 21 lymphoblastoid cell lines (Figure 1). Specifically, in terms of leukocyte endothelial adhesion the Ramos line revealed 5% of cells remaining whereas over 35% of the Trisomy 21 cells were remaining following three washes. Normal lymphoblastoid cell lines fell in the middle of this range. Peripheral blood mononuclear cells revealed 5–10% adhesion, consistent with prior reports [10], [16], [17].

### Inter-individual Variation in Leukocyte Endothelial Adhesion

**Figure 2:**
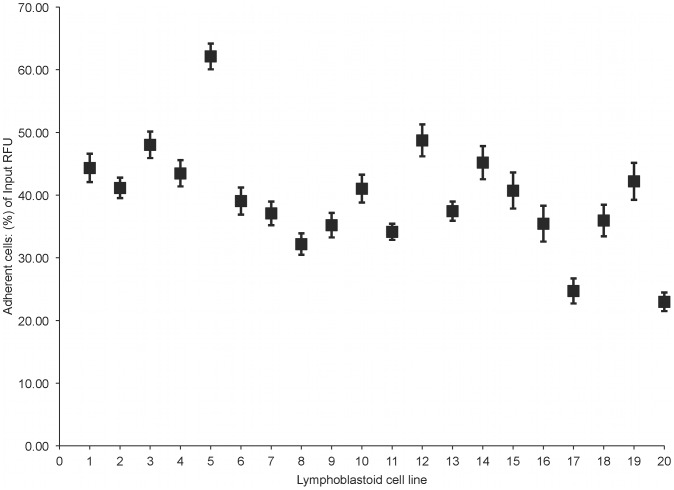
Variations in leukocyte endothelial adhesion among 20 selected unrelated lymphoblastoid cell lines. Leukocyte endothelial adhesion was assessed in 20 unrelated lymphoblastoid cell lines. For each cell line, 8 technical replicates were obtained per plate. The figure reveals inter-lymphoblastoid cell line differences in leukocyte endothelial adhesion with a range from 22.42–61.12% input RFU, a mean of 40.25% (std. dev. ±10.37), and a median of 39.80%. Minimal variability is evident in the assay with a CV of less than 10% (CV = 8.68, Z′-factor = 0.67, SNR = 18.41).

Using twenty unrelated lymphoblastoid cell lines generated from normal subjects, we measured their leukocyte endothelial adhesion in order to estimate the inter-individual variability obtained through our high-throughput assay. Figure 2 reveals the range of adhesion in normal lymphoblastoid cell lines. Specifically the range was from 22.42–62.12% input RFU with a mean of 40.25% (std. dev. ±10.37), and a median of 39.80%.

### Heritability of Leukocyte Endothelial Adhesion

**Figure 3:**
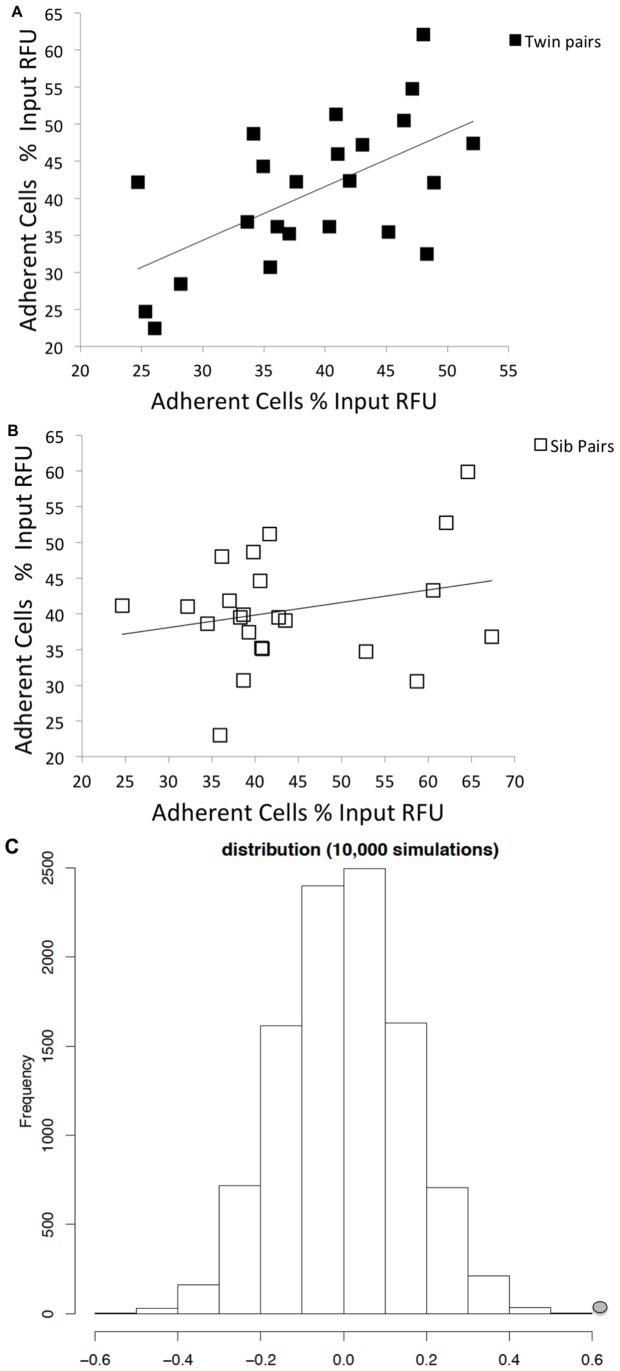
Intra-pair similarity for leukocyte endothelial adhesion is greater in monozygotic twins than sibling pairs. Figure 3A shows the correlation in adhesion of 23 monozygotic twin pairs compared to 23 matched sibling pairs (Figure 3B). Axes represent the % of adherent cells based on RFU, with each axis corresponding to one of the two subjects for a given pair. A higher mean correlation is seen in the identical twins (_ICC_MZ 0.60) as compared to the siblings (_ICC_SIB 0.25). The resultant heritability had an empirical p-value<0.0001 based on 10,000 simulations by randomizing the samples to generate a null distribution (Figure 3C). The round dot represents the observed heritability value from our study.

We were interested to assess the extent to which genetic factors contributed to inter-individual differences in leukocyte endothelial adhesion. We performed a heritability study for leukocyte endothelial adhesion utilizing lymphoblastoid cell lines derived from identical twins and those derived from matching sibling pairs. The hypothesis was that there would be greater correlation between the adhesion of the identical twins than that of the sibling pairs. Summarized subject data are reported in Table S1. Figure 3 reveals that this was indeed the case. Figure 3 demonstrates the leukocyte endothelial adhesion for each pair of lymphoblastoid cell lines. A higher correlation in adhesion was observed between the twins (Fig. 3A) than in the siblings (Fig. 3B) with an intra-pair similarity for leukocyte endothelial adhesion in monozygotic twins of 0.60 compared to 0.25 in the sibling pairs. The extent of heritability is essentially an extension of the excess correlation seen in monozygotic twins versus sibling pairs. We quantified the extent to which these differences are attributable to underlying genetic factors [11], [12] and found the heritability of leukocyte endothelial adhesion to be 69.66% (p-value<0.0001) (Fig. 3C), meaning that essentially 70% of the variability in leukocyte endothelial adhesion between individuals can be attributed to genetic factors.

### Role of confounding factors

**Figure 4:**
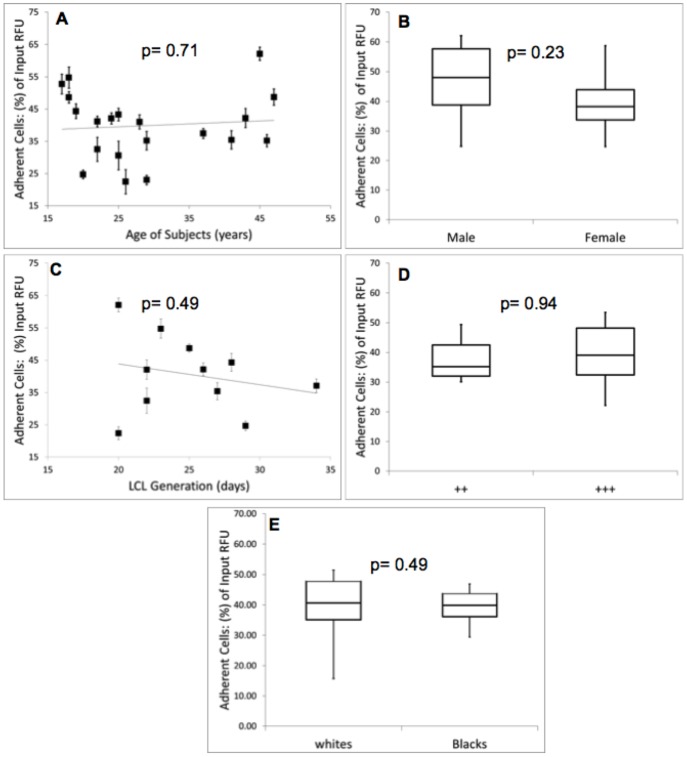
Impact of confounding factors on leukocyte endothelial adhesion among 20 unrelated lymphoblastoid cell lines. In 4A, we explored the role of subject age on leukocyte endothelial adhesion. No correlation was observed (p-value = 0.77). Figure 4B stratifies adhesion by gender. As found in prior studies leukocyte endothelial adhesion is slightly decreased in women, although this was not statistically significant in our cohort (p-value = 0.23). In figure 4C we determined if the duration of lymphoblastoid cell line generation from peripheral blood mononuclear cell affected the adhesion and found no correlation. In 4D we assessed the impact of cell culture growth on adherence. Lymphoblastoid cell line slow culture growers with a doubling time of seven days are represented by ++ and fast culture growers with a doubling time of 3 days are represented by +++. We found that cell cutlure growth conditions did not affect adhesion (p-value = 0.85). In figure 4E we assesed the effect of ethnicity on LEA and observed that ethnicity had no effect on LEA (p-value = 0.49).

It is certainly possible, though, that confounding factors contribute to differences in leukocyte endothelial adhesion between lymphoblastoid cell lines. In Figure 4, we sought to identify non-genetic factors that could potentially confound our analysis. We assessed whether or not there was any correlation of leukocyte endothelial adhesion for 20 unrelated individuals with subject age (Figure 4A), gender (Figure 4B), cell growth rate (Figure 4C) transformation time (Figure 4D) and ethnicity (Figure 4E). Subject age (p-value = 0.71), gender (p-value = 0.23), transformation time (p-value = 0.49), cell growth rate (p-value = 0.94) and ethnicity (p-value = 0.49) all had no correlation with endothelial adhesion. Sample constraints prevented matching the two groups exactly based ethnicity. There is a suggestion in the literature that there may be some variation in adhesion based on ethnicity [18], [19]. In hindsight, this observation is not surprising in light of our heritability analysis as ethnicity is a crude measure of genetic background. In this study we observed no difference in mean adhesion between white and Black subjects. More importantly, from the standpoint of confounding there was also no ethnic based difference in the variance of adhesion (p-value = 0.57). A literature search did not reveal any other covariates known to influence leukocyte endothelial adhesion.

## Discussion

The leukocyte endothelial adhesion assay we developed is able to sensitively and reproducibly detect differences in leukocyte adhesion to endothelial cell monolayers. This novel high-throughput assay was able for the first time to reveal the extent to which the important biological property of leukocyte endothelial adhesion is impacted by genetic variation.

Lymphoblastoid cell lines have evolved into a powerful model system for functional population-based studies in humans. Lymphoblastoid cell lines are transformed cell lines derived from a subject's circulating B-lymphocytes. They were originally generated as a perpetual resource of a specific subject's DNA. Given the associated difficulties with maintenance, storage, collection, and the control of confounding variables in clinical samples, they have evolved into a popular model system [20], [21]. In large part this trend is due to the fact that lymphoblastoid cell lines to date are the only feasible and cost-effective method to conduct functional, cellular or molecular assays in large population-based human studies. As a cell line, environmental factors can be controlled between lines, which removes many confounders that are routinely found in clinical samples. Hence, transformation of clinical samples into lymphoblastoid cell lines provides convenience, replicability, and environmental uniformity.

In this study, we sought to explore the feasibility of using lymphoblastoid cell lines to assess the heritability of leukocyte endothelial adhesion. It was first necessary to create a high-throughput assay for leukocyte endothelial adhesion that would enable us to compare the adhesion of multiple different cell lines. We developed a robust assay (Z′-factor = 0.67). The excellent Z′-factor suggests that the variability introduced by the washing step in other high-throughput assays was not an issue in our assay [22].

To our knowledge the present paper is the first study to determine the heritability of leukocyte endothelial adhesion. Current approaches have the power to reveal the genetic network underlying this trait. For these genetic tools to work, though, there must be a heritable basis to leukocyte endothelial adhesion. A substantial body of literature implicates genetic variation as a basis for differential leukocyte adhesion [7], [8] in conditions that include chromosomal alterations [10], Mendelian conditions [9], and complex disease [23]. In this study, we quantitate the extent that genetic factors contribute to inter-individual variability in leukocyte endothelial adhesion. By comparing leukocyte endothelial adhesion between monozygotic twins and matched sibling pairs, we found that 70% of the variation in leukocyte endothelial adhesion is due to underlying genetic factors.

The use of lymphoblastoid cell lines is a potential limitation of this study, though. An issue regarding the use of lymphoblastoid cell lines is the extent to which they serve as relevant surrogates for different primary tissues. Previous work has demonstrated the broad translatability of studies with lymphoblastoid cell lines [24], in particular for complex traits in which white blood cells are the most relevant human tissue as in leukocyte endothelial adhesion or the diseases of type 1 diabetes, Crohn's disease, and multiple sclerosis [25], [26]. While endothelial cells are important in mediating leukocyte endothelial adhesion and there are unique molecular features specific to endothelial cells [27], [28], it is their interaction with white blood cells that results in the leukocyte endothelial adhesion phenotype [29]. Hence, it is likely that many of the genetic factors influencing leukocyte endothelial adhesion can be identified with lymphoblastoid cell lines alone.

A persistent concern about the usefulness of lymphoblastoid cell lines is that changes associated with the Epstein-Barr virus transformation reduce their value and relevance as a model. The virus transformation of primary white blood cells does change some of the cellular properties within the lymphoblastoid cell lines [30]. The Epstein-Barr virus transformation induces a shift in white blood cells to an activated state [31], [32]. Caliskan et al. [30] identified an enrichment of gene expression for the pathways of transcription regulation, cell cycle control, and immune response in the lymphoblastoid cell lines. A standard component of leukocyte endothelial adhesion assays is the external activation of endothelial cells with agents such as interleukin-1β or TNF-α to facilitate leukocyte adhesion. In our studies, exogenous addition of these agents did not improve lymphoblastoid cell line adhesion, likely due to the activated state of the lymphoblastoid cell lines [33]. There is also concern that over time lymphoblastoid cell lines may change cellular properties. In studies by Im et al. of 540 HapMap lymphoblastoid cell lines, differences in cellular growth rate were observed when lymphoblastoid cell line populations that were obtained at different times were compared [34]. It is possible that there may be age and generation dependent effects on activity or expression of cellular mediators with lymphoblastoid cell lines. We did not find adhesion to be confounded by lymphoblastoid cell line-specific factors.

Adhesion in lymphoblastoid cell lines demonstrates similar variation observed in primary leukocytes. In our experiments, we observed, that the Burkitt lymphoma cell lines show the least endothelial adhesion while Trisomy 21 lymphoblastoid cells bind the highest compared to the lymphoblastoid cell lines derived from 92 normal subjects. A study comparing the expression of adhesion molecules as determined by flow cytometry in lymphoblastoid cell lines generated from normal peripheral blood mononuclear cells and Burkitt lymphoma cell lines demonstrated that the normal lymphoblastoid cell lines express higher levels of integrins, including integrin alpha 4 beta 1, leukocyte function associated antigen LFA-1, and integrin alpha 5 beta 3 as well as enhanced expression of the IgG superfamily adhesion molecules, like ICAM-1, and leukocyte function associated antigen type 3, and, glycoproteins like L-selectin and CD34 [8]. This is consistent with our observation of less adhesion of Burkitt lymphoma cell lines than the normal lymphoblastoid cell lines. The increased adhesion seen in Down's syndrome is thought to be due to over-expression of the CD18 integrin found on chromosome 21 [10]. Thus, lymphoblastoid cell lines appear to maintain the majority of factors that influence the unique endothelial adhesion of their primary leukocytes.

Another potential limitation is the use of an immortalized endothelial cell line. EA.hy926 is one of the best-characterized and most frequently used endothelial cell lines [35]. It is a product of the fusion of human umbilical vein endothelial cells and human lung carcinoma line A549. It is contact inhibited in growth and expresses von Willebrand factor. It is a transformed endothelial cell line that expresses intercellular adhesion molecule 1, vascular cell adhesion molecule 1, and E-selectin [36]. Given its similar leukocyte binding properties to human umbilical vein endothelial cells, EA.hy926 is frequently used in adhesion assays [37]. EA.hy926 has the distinct advantage for this study of homogeneity unlike human umbilical vein endothelial cells, or other primary cell lines, which are harvested from multiple donors [37] and suffer from lot to lot variability. The introduction of such variability would be problematic for a genetic study of this nature.

There are several strengths of this study. The assay was implemented on a high- throughput platform with very little intra-individual or inter-plate variability. Access to a large cohort of monozygotic twin and sibling pair lines enabled us to confidently identify leukocyte endothelial adhesion as a heritable trait. Heritability is the best statistic for the representation of the genetic contribution to phenotypic variance [38]. In particular, the use of identical twins and sibling pairs allows one to assume that the total variation introduced by the common environment shared by family members is equal [38]. We were able to exclude systematic underlying confounding factors including age, gender [39], ethnicity [18], [19] and cell culture conditions [31], [40]. Finally, while there have been many other studies that have obliquely suggested the heritability of this trait, this is the first one to our knowledge that was designed to directly assess and quantify it.

In summary, there is a major heritable component to leukocyte endothelial adhesion. Genetic predisposition appears to play a significant role in inter-individual variability of leukocyte endothelial adhesion. As such, future genetic studies should be able to identify those key molecular elements that regulate this important cellular trait.

## Supporting Information

Figure S1 **Standard curves for cell lines.** Four parametric logistic curves of (A) Trisomy 21 lymphoblastoid cell line (B) Ramos Burkitt lymphoma (C) GM14581 a reperesentative twin lymphoblastoid cell line (D) U937 a monocytic leukemic cell line (E) and peripheral blood mononuclear cells (PBMC) were generated using known concentration Calcein AM labeled cells. On the x-axis is the known concentration of cells lines and on the Y- Axis are the relative fluorescence units. The standard curve generated was used to deduce the adherent cells that remained following washes.
(TIFF)

Table S1 **Subject Characteristics.**
(PDF)
