# Supplementary figures and images for: Genetic Variation Is the Major Determinant of Individual Differences in Leukocyte Endothelial Adhesion
Source: PLoS One. 2014 Feb 10;9(2):e87883. doi: 10.1371/journal.pone.0087883 (PMC3919726; doi:10.1371/journal.pone.0087883)

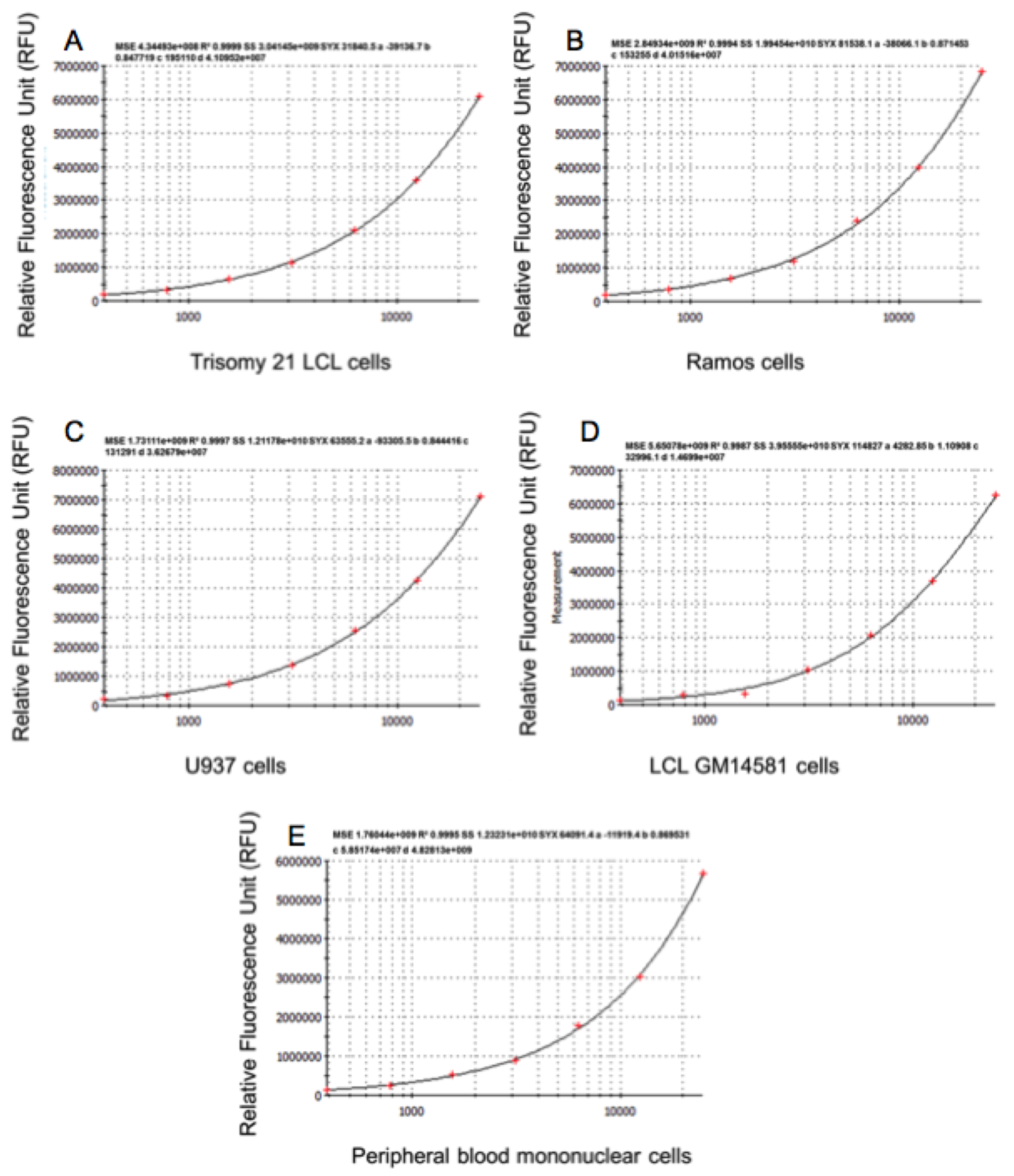

Supplement: Figure S1 — Standard curves for cell lines. Four parametric logistic curves of (A) Trisomy 21 lymphoblastoid cell line (B) Ramos Burkitt lymphoma (C) GM14581 a reperesentative twin lymphoblastoid cell line (D) U937 a monocytic leukemic cell line (E) and peripheral blood mononuclear cells (PBMC) were generated using known concentration Calcein AM labeled cells. On the x-axis is the known concentration of cells lines and on the Y- Axis are the relative fluorescence units. The standard curve generated was used to deduce the adherent cells that remained following washes. (TIFF) [file pone.0087883.s001.tiff]
