# Supplementary material for: Genetic Variation Is the Major Determinant of Individual Differences in Leukocyte Endothelial Adhesion
Source: PLoS One. 2014 Feb 10;9(2):e87883. doi: 10.1371/journal.pone.0087883 (PMC3919726; doi:10.1371/journal.pone.0087883)
Supplement: Table S1 — Subject Characteristics. (PDF) [file pone.0087883.s002.pdf]

	Twins	Siblings
Number of cell lines	46	46
Gender	12 Male; 34 female	12 Male; 34 Female
Age	33.43±13.20	24.41 ± 4.67
